# Toddlers’ diurnal cortisol levels affected by out-of-home, center-based childcare and at-home, guardian-supervised childcare: comparison between different caregiving contexts

**DOI:** 10.1007/s00787-019-01432-3

**Published:** 2019-11-08

**Authors:** Katja Tervahartiala, Linnea Karlsson, Juho Pelto, Susanna Kortesluoma, Sirpa Hyttinen, Annarilla Ahtola, Niina Junttila, Hasse Karlsson

**Affiliations:** 1 The FinnBrain Birth Cohort Study, Turku Brain and Mind Center, Department of Clinical Medicine, University of Turku, Lemminkäisenkatu 3, 20520 Turku, Finland; 2 Department of Child Psychiatry, Turku University Hospital and University of Turku, Kiinamyllynkatu 4-8, 20520 Turku, Finland; 3 Institute of Biomedicine, University of Turku, Kiinamyllynkatu 10, 20520 Turku, Finland; 4 Finnish Institute of Occupational Health, Topeliuksenkatu 41a B, 00250 Helsinki, Finland; 5 Department of Psychology and Speech-Language Pathology, University of Turku, Assistentinkatu 7, 20500 Turku, Finland; 6 Department for Teacher Education, University of Turku, Assistentinkatu 5, 20500 Turku, Finland; 7 Department of Psychiatry, Turku University Hospital and University of Turku, Kiinamyllynkatu 4-8, 20520 Turku, Finland

**Keywords:** Hypothalamus–pituitary–adrenal (HPA) axis, Diurnal cortisol levels, Out-of-home, center-based childcare, At-home, guardian-supervised childcare, Early childhood education and care (ECEC)

## Abstract

Previous research suggests that attending non-parental out-of-home childcare is associated with elevated cortisol levels for some children. We aimed to compare diurnal saliva cortisol levels between children having out-of-home, center-based childcare or those having at-home, guardian-supervised childcare in Finland. A total of 213 children, aged 2.1 years (SD = 0.6), were drawn from the ongoing Finnish birth cohort study. Saliva samples were collected over 2 consecutive days (Sunday and Monday), with four samples drawn during each day: 30 min after waking up in the morning, at 10 am, between 2 and 3 pm, and in the evening before sleep. These results suggest that the shapes of the diurnal cortisol profiles were similar in both childcare groups following a typical circadian rhythm. However, the overall cortisol levels were on average 30% higher (95% CI: [9%, 54%], *p* = .004) with the at-home childcare in comparison with the out-of-home childcare group. Furthermore, a slight increase in the diurnal cortisol pattern was noticed in both groups and in both measurement days during the afternoon. This increase was 27% higher ([2%, 57%], *p* = .031) in the out-of-home childcare group during the out-of-home childcare day in comparison with the at-home childcare day. The elevated afternoon cortisol levels were partly explained by the afternoon naps, but there were probably other factors as well producing the cortisol rise during the afternoon hours. Further research is needed to define how a child’s individual characteristic as well as their environmental factors associate with cortisol secretion patterns in different caregiving contexts.

## Introduction

The majority of children in Western societies participate in center-based, out-of-home childcare [1]. As out-of-home childcare is also part of the educational program in many countries, previous research studies have suggested that out-of-home childcare has many positive effects on children’s socio-emotional development, cognitive skills, and educational outcomes [2, 3]. Alongside with the possible advantages, earlier research studies have also shown that attending non-parental, out-of-home childcare is associated with elevated cortisol levels for some children [4, 5]. In out-of-home childcare settings, young children may need to cope in a different caregiving environment; with parental separation; and with interactions with multiple adults and peer relations, which may involve emotional arousal. These issues have been considered to be among the stressors affecting children’s cortisol levels in an out-of-home childcare context [6, 7].

Vermeer and van IJzendoorn [8] reviewed nine studies, where children’s cortisol levels with out-of-home childcare were analyzed. The main finding was that children displayed higher cortisol levels with out-of-home childcare compared to the days they spent at home. The effect was more notable in toddlers younger than 36 months. In seven studies, the difference between the sexes was analyzed, but sex was not related to cortisol levels. These findings are well replicated in later research studies suggesting that in the out-of-home childcare context, children have a cortisol increase particularly from mid-morning to mid-afternoon compared to the typical decline over the course of the day in an at-home, guardian-supervised setting [9–13]. Elevated cortisol levels were especially notable in children, who had full-time or full-day, out-of-home, center-based childcare in comparison with those children who had a part-time or a half-day childcare [14]. An increased cortisol pattern in children has been observed also during the transition to out-of-home childcare from at-home, guardian-supervised childcare [10].

Nevertheless, it is not entirely clear which components in professional out-of-home childcare influence children’s physiological responses. Previous research suggests that the childcare quality, the caregiver–child interaction, and child characteristics as well as peer relations and quantity of childcare are among the key elements contributing to the variation in stress regulation responses among children in out-of-home childcare [7]. However, it is not known which of the observed factors are related to the childcare setting as such, as earlier research studies did not include an at-home childcare comparison group, instead, in all the studies, the same child was assessed during their out-of-home childcare day and during their at-home day. To our knowledge, there are no earlier studies assessing toddlers’ diurnal cortisol levels in the childcare context involving a comparison group of separate children, who did not attend any type of out-of-home, childcare groups.

The hypothalamus–pituitary–adrenal (HPA) system produces the stress hormone cortisol in response to psychological or physical stress to mobilize energy and facilitate physical responses to potential threats. Long-term exposure to stress during childhood may be a risk factor for a child’s socio-emotional and cognitive development and also may undermine the functioning of the immune system and increase the child’s vulnerability to stress-related illnesses [15–17]. Of note, there is evidence showing that low and hyporeactive cortisol profiles in response to stressors may also be associated with adverse developmental outcomes, such as disruptive behavior disorders [18]. Hence, both too low and too high cortisol levels may imbalance homeostasis, cause an allostatic load, and associate with later health outcomes [19].

The circadian rhythm typical for cortisol secretion in adults (i.e., cortisol levels are highest after waking up and decline over the later daytime and evening hours) has been observed in children starting from as early as 3 months of age depending on the study [16]. Cortisol awakening response (CAR) defines the period of the cortisol peak occurring 30–45 min after awakening. Previous research suggests that the CAR can be observed consistently throughout the toddler and childhood years [20]. Most toddlers take naps, and napping is shown to influence the diurnal cortisol pattern. Thus, cortisol secretion does not only follow the circadian rhythm but is also nap-dependent in toddlers. The pronounced cortisol rise is observed to follow both the morning and afternoon naps in comparison with the no-nap condition [21].

This study aimed to compare diurnal saliva cortisol levels between toddlers in an out-of-home, center-based childcare environment with a separate group of toddlers being cared for at their own home in Finland. The out-of-home childcare group of the children at either private or public childcare centers followed the early childhood education and care (ECEC) program criteria. The at-home childcare group had children cared for at home by their mother, father, or another caregiver that the child was familiar with. The Ministry of Education and Culture is responsible for the overall planning, guidance, and monitoring of the ECEC in Finland. A personal ECEC plan is drawn up for each participating child, and it consists of the objectives to support child's development, learning, and the need for special support if necessary [22]. The child has a legal right from an early age for full-time ECEC if the parents work or study full time, or if it is best for child’s development. Otherwise, participation is limited to 20 h per week. The personnel are qualified, and at least one staff member per group in center-based care must have a Bachelor’s level university education in pedagogics. The legislation also sets out requirements for group sizes and child-to-caregiver ratios. The fees are rather low in comparison with many other European countries. In Finland, the monthly fee depends on the household’s income, and participation is free of charge for the low-income families [23].

Our study covered four saliva cortisol measurements during 2 consecutive days, which enabled us to explore diurnal cortisol patterns with the baseline levels on Sunday, with Sunday being an at-home childcare day for both groups of the children. Previous research studies have mostly covered two or three measurements during each day, which may have limited the analyses of the diurnal cortisol profiles [12].

Based on earlier literature, we created four hypotheses being: (1) the overall cortisol levels would be higher in the out-of-home, childcare group than in the at-home childcare group; (2) in the out-of-home, childcare group, the afternoon cortisol levels would be higher in the childcare day (Monday) than during the at-home day (i.e., Sunday); (3) the duration of childcare attendance, larger group size in childcare, and full-time versus part-time childcare would be associated with higher overall cortisol levels in this group; and (4) in the at-home childcare group, the afternoon cortisol levels would not differ between the 2 days (i.e., Sunday and Monday).

## Methods

### Participants

The participants were drawn from the larger FinnBrain Birth Cohort Study (*N* = 3808), which is a population-based pregnancy cohort with aims to identify biomarkers related to prenatal stress and early life stress exposure as well as trajectories for common psychiatric and somatic illnesses. Recruitment took place during the first ultrasound visit during gestational week (gwk) 12 by research nurses in Southwest Finland and the Åland Islands. According to the study inclusion criteria, the nurses approached families with a sufficient knowledge of Finnish or Swedish and selected children with a normal ultrasound screening result [24].

Research recruitment for this study was carried out through personal contact by research personnel between April 2014 and July 2017 (all months of the year included). According to the eligibility criteria, the child needed to have either out-of-home, center-based childcare or else have at-home childcare by a guardian or another caregiver that the child was familiar with. Other forms of childcare (e.g., home-based childcare, which is childcare operated in small groups in someone’s home or 24-h out-of-home childcare services) were excluded. After selecting the children, the respective childcare centers were recruited. The research permit was obtained from three municipalities from southwest Finland, and the study was conducted in urban and suburban areas of these municipalities. A total of 32 childcare centers participated, of which, 14 were private, and 18 were public childcare units.

**Fig. 1:**
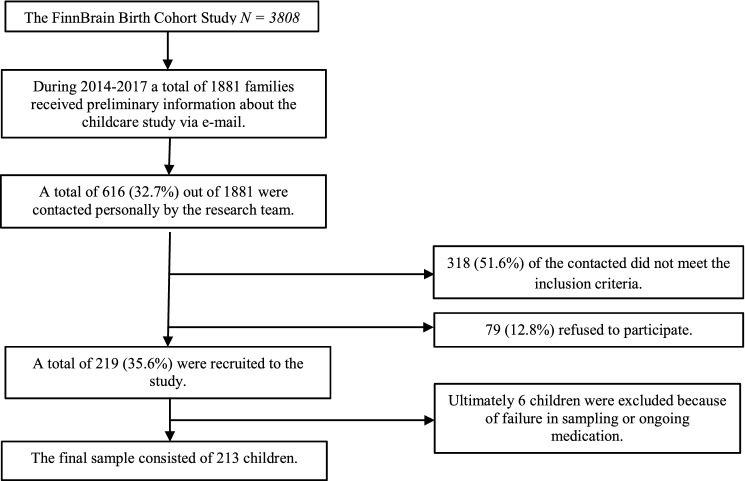
Flowchart of the recruitment process

The recruitment process is illustrated in Fig. 1. First, all the FinnBrain Birth Cohort families (*N* = 1881), whose child was at an appropriate age for the study and lived in the research area during that time period, were approached by e-mail and given preliminary information about the current project. To facilitate the logistics of data collection, we wanted to restrict the numbers of childcare centers. Thus, the recruitment process was focused on selected geographical areas, which however were diverse and served as a representation of the whole birth cohort population.

A total of 616 families were personally contacted by the research team to sort out their eligibility to participate in the research. In all, a total of 318 of those families contacted did not meet the inclusion criteria, and 79 families refused to participate. In the end, a total of 219 toddlers, of which 109 were attending an out-of-home, center-based childcare group and 110 toddlers who were in at-home childcare group, were recruited. Ultimately, six children were excluded because of a failure in the saliva sampling, or because the children’s mothers reported their children to be taking ongoing medications or had diseases possibly affecting the children’s cortisol levels. The final sample consisted of 213 children, of which, 106 belonged to the out-of-home childcare group, and 107 were in at-home childcare group.

### Diurnal cortisol collection

Saliva cortisol was used to measure the diurnal cortisol levels of the toddlers. Saliva samples from each child were collected over 2 days. Four samples were drawn each day: 30 min after waking up in the morning, at 10 am, between 2 and 3 pm, and in the evening before sleep. The timing of the sample collection was designed to cover a full day from awakening until bedtime on both the out-of-home childcare days and the at-home childcare days. Two samples were collected during the out-of-home childcare day to analyze the difference between mid-morning (10 am) and mid-afternoon (2–3 pm) cortisol values. Most parents picked up their children from the childcare centers at the latest between 4 and 5 pm, and the sample collection was implemented before that. Some children had part-time care, which is defined as a limited number of childcare days during a month. However, the children in part-time care still attended a full day during their childcare days enabling both morning and afternoon saliva sample collection to take place in the out-of-home childcare center in a manner identical to those attending full-time care.

The first day of collection was Sunday, when all the children were at home, and the second day was Monday, when the children were attending an out-of-home, childcare group or stayed at home for care, according to their allocation. For eight children, samples were not taken on the protocol days, because the children never attended out-of-home childcare on Mondays. However, the samples were collected at the childcare center immediately after the day off. Parents collected saliva samples at home, and childcare personnel collected samples at the childcare center. Sample taking was taught personally to the caregiving personnel by the research nurse and completed by giving written instructions and information and a related tutorial video.

The saliva samples were collected using Salimetrics infant swabs (www.salimetrics.com) keeping the polymer swab in the child’s mouth for 2 min during the collection. Parents and childcare personnel were advised to avoid having the children do physical activity for 30 min and to let the children eat 15 min before sampling.

### Sample storage

Saliva samples were placed in swab storage tubes and kept in a refrigerator from 2 to 5 days between sample taking and the delivery to the research center. An interlaboratory stability test for cortisol in saliva verified that the samples remained stable at room temperature for at least 7 days, and storage did not have an effect on the measurement [25]. For most of the study period, samples were collected directly from homes and childcare centers by research assistants, but a small number of samples, at the beginning of the study, were returned by mail within 5 days of collection. After delivery, the saliva samples were immediately centrifuged (4 °C, 15 min, 1800×*g*) and frozen at − 70 °C. The samples were analyzed at The Finnish Institute of Occupational Health, Helsinki, Finland, which conducted the international quality controls performed for the method. The free cortisol in saliva was analyzed using a cortisol saliva luminescence immunoassay (RE62111, IBL, International, Germany). The linear reportable range of the assay was 0.30–86.5 nmol/l. The coefficient of variation for the intra- and inter-assay of the method was 5 and 8%, respectively.

### Background data

The background data of the mothers (i.e., age, education, income level, origin, language, and the duration of pregnancy) were determined from the cohort research questionnaires during the pregnancy and the Medical Birth Register of the Finnish National Institute for Health and Welfare (www.thl.fi). Parents filled in a form describing their child’s childcare history and daily rhythm, such as the child’s waking time in the morning and their time of afternoon naps, their sleeping time during the evening, and their mealtimes as well as illnesses and prescribed medication on the saliva collection days. Childcare personnel filled out corresponding information about sleeping and mealtimes during the childcare day.

### Statistical analyses

We used the time since wake up (= cortisol measurement time—wake-up time in the morning, measured in hours) as our sample time variable, because the cortisol values began to decline about 30–45 min after waking up in the morning. The families were instructed to take samples 30 min after awakening, but the sampling time varied a little between the children and, as a consequence, we calculated time since wake up for each participating child. There were some missing cortisol measurement time values (*N* = 2–10) at each measurement point in the records. The missing time values were imputed by the median measurement time (since wake up) in that measurement point. Furthermore, we used base10 logarithm-transformed saliva cortisol values, because the distribution of the original values was strongly positively skewed.

The children’s saliva cortisol levels were then modeled using a linear mixed effects model. We were mostly interested in how the diurnal cortisol levels differed between the groups (i.e., in an out-of-home, childcare group or in an at-home childcare group) and also between the days (Sunday or Monday). Therefore, we included the binary variables group and day in our model. The diurnal cortisol profile, i.e., the relationship between cortisol and time (since wake up) was modeled by a natural cubic spline [26] (with cutoff points at 2 h and 44 min and 7 h and 10 min, i.e., at the median time values at the second and third time points). Furthermore, we controlled for maternal education level (e.g., high school/vocational education, polytechnics/applied university, or university degree), maternal income level (<1500 eur, 1500–2500 eur, > 2500 eur), the age of the child (in years), and the sex of the child. Lastly, instead of anchoring the afternoon sampling with the nap, the effect of afternoon naps on the afternoon measurements was controlled for by a three-class variable with possible values “ < 15 min,” “between 15 and 60 min,” and “over 60 min/no naps” indicating how long after waking up from the nap the sample was taken. This was chosen because the timing of the nap varied between children and not all the children took a nap. A total of 62.3% of the children in the out-of-home childcare and 72% of the children in the at-home childcare group took naps on Sunday. Correspondingly, a total of 88.7% of the children in the out-of-home childcare and 71% of the children in the at-home childcare group took naps on Monday.

The child-specific effects (i.e., the random effects) used in the model were chosen by testing a few sensible options and then choosing the model that had the lowest Akaike information criterion (AIC) [27]. By this means, a result was achieved that included in the model a linear time effect (i.e., random slope) and separate random intercepts for Sunday and Monday for each child.

We then fitted the model with only main effects to find out how the cortisol levels differed between the groups (Hypothesis 1) and how they were associated with the other above-mentioned variables. After this, we examined in each group whether there were differences in the afternoon (defined as 7 h and 10 min since wake up, i.e., the median afternoon measurement time) cortisol levels between Sunday and Monday (Hypotheses 2 and 4). This was done by fitting a model including the interactions among the group, the day and time spline terms (in addition to the above-mentioned main effects), and then using the model to estimate the cortisol differences at 7 h and 10 min since wake up. The standard errors for these differences were estimated by bootstrapping the model (using 1000 bootstrap samples). Finally, we fitted the main effects model for only the out-of-home childcare group also including in the model the variables for the duration of childcare attendance, the group size in childcare center, and the childcare form (part-time vs. full-time) to test how these variables were associated with the cortisol levels (Hypothesis 3).

As some of the children had exceptionally high cortisol levels, we also fitted the main effects models using robust regression analysis. Robust regression gives, in practice, less weight to the extreme observations, and it therefore gives results that are less sensitive to the effect of some of the extreme observations. Random intercept was the only random effect used in the robust regression models.

**Fig. 2:**
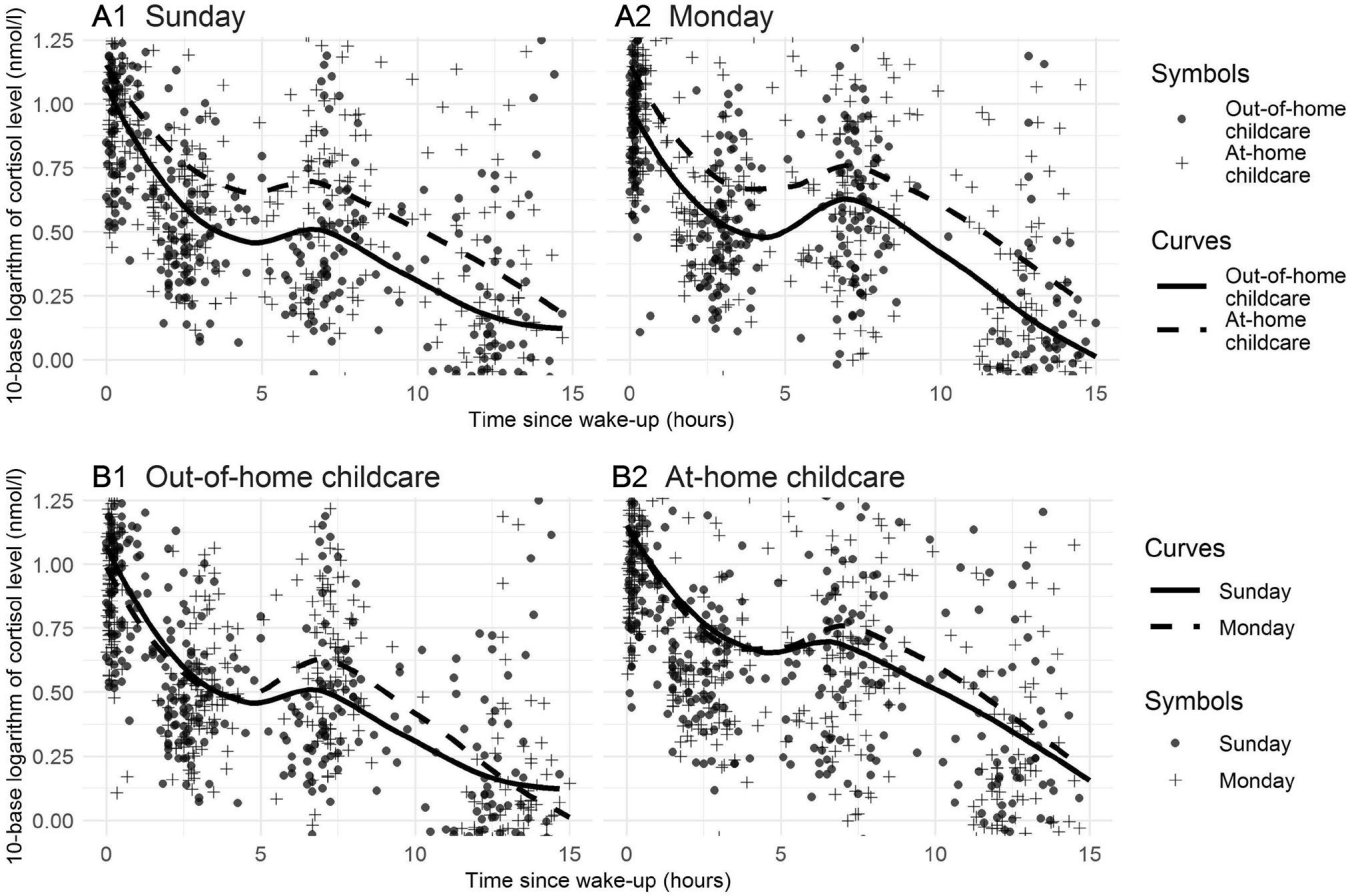
Logarithm of the cortisol values plotted against time since wake up for each group and day. The curves are LOESS smoothing curves based only on the time since wake up, group, and day (i.e., they are not predictions from our mixed effects models). Note: the highest and lowest cortisol values are cut out from the figures to make the diurnal cortisol profile appear more clearly

All the analyses were performed in R [28] with the following packages: nlme [29] for fitting the mixed models, robustmll [30] for robust analysis, and ggplot2 [31] for Fig. 2.

## Results

### Sample characteristics

Table 1 summarizes the characteristics of the participants. All the participants were ethnically Caucasian, and the mother’s native language was primarily Finnish. The proportion of boys and girls was in balance between the groups, but the mean age of the children was higher in the out-of-home, childcare group being 2.26 (SD = 0.6) years, while in the at-home childcare group, the mean age was 2.00 (SD = 0.5) years. The duration of out-of-home childcare attendance varied amongst the children with some who had attended for less than 2 weeks and others for 2 years with a mean of 9.65 months (SD = *7.3*). Most children (*N* = 76) participated in full-time childcare, and a smaller number of the children (*N* = 30) had part-time childcare. The full-time childcare included, on average, 20 childcare days within a month, and part-time childcare consisted of maximum 16 days (*N* = 24) or 11 days (*N* = 6) within a month. All the childcare centers followed the ECEC program criteria, and the participating children had a similar schedule within centers. The average group size in the childcare centers was 13.47 (SD = 3.8) children, and the child-to-caregiver ratio was on average 4.59 (SD = 1.2), while in the at-home childcare group, the child-to-caregiver ratio was only 1.78 (SD = 0.8). The main caregiver in the at-home childcare group was a mother (*N* = 91) and for only a small number of participants the caregiver was a father (*N* = 10) or another caregiver (*N* = 6) at home. More than half (57%) of the children in the at-home childcare also had siblings at home concurrently, and the number of the siblings ranged between one (41.1%), two (11.2%), or three (4.7%) siblings during the study participation.

**Table 1:** Demographic characteristics of the participants

	Out-of-home childcare	At-home childcare	Study sample	*p*
*N*	106	107	213	
Child characteristics				
Age (years), mean (SD)	2.26 (0.6)	2.00 (0.5)	2.13 (0.6)	0.001
Male, *N* (%)	63 (59.4%)	53 (49.5%)	116 (54.5%)	0.147
Child-to-caregiver ratio, mean (SD)	4.59 (1.2)	1.78 (0.8)		
Duration of childcare attendance (months), mean (SD)	9.65 (7.3)			
Full-time care, *N* (%)	76 (71.7%)			
Group size in childcare centers, mean (SD)	13.47 (3.8)			
Number of siblings at home, mean (SD)		0.78 (0.8)		
Maternal characteristics				
Age (years), mean (SD)	34.10 (4.1)	34.08 (4.5)	34.09 (4.3)	0.97
Maternal education, *N* (%)				
High school/vocational education	16 (15.1%)	29 (27.1%)	45 (21.1%)	0.048
Polytechnics/Applied University	31 (29.2%)	34 (31.8%)	65 (30.5%)	
University degree	59 (55.7%)	44 (41.1%)	103 (48.4%)	
Maternal income, *N* (%)				
Low < 1500 eur	36 (33.9%)	42 (39.3%)	78 (36.6%)	0.145
Med 1501–2500 eur	55 (51.9%)	58 (54.2%)	113 (53.1%)	
High > 2501 eur	15 (14.2%)	7 (6.5%)	22 (10.3%)	
Mother’s origin, *N* (%)^a^				
Finnish	100 (99.0%)	103 (98.1%)	203 (98.5%)	0.584
Other	1 (1.0%)	2 (1.9%)	3 (1.5%)	
Mother’s native language, *N* (%)^b^				
Finnish	97 (96.0%)	102 (98.0%)	199 (97.1%)	0.237
Swedish	4 (4.0%)	1 (1.0%)	5 (2.4%)	
Other		1 (1.0%)	1 (0.5%)	
Duration of pregnancy (weeks), mean (SD)	39.67 (2.0)	39.75 (1.6)	39.71 (1.8)	0.752

*P* values based on *t *test for age; child-to-caregiver ratio; duration of pregnancy; and *χ*^2^ test for gender, education, income, origin, and language

^a^Based on *N* = 101 for out-of-home childcare and *N* = 105 for at-home childcare

^b^Based on *N*  = 101 for out-of-home childcare, and *N*  = 104 for at-home childcare

The mother’s age, income level, and duration of pregnancy were similar in both groups, but maternal education was lower in the at-home childcare group. However, it should be noted that the overall education level was high in the whole study sample, as 48.8% of the mothers had a university degree education.

The descriptive statistics of the saliva cortisol sampling, including sampling times, waking times, mealtimes, and the median of raw cortisol values are, respectively, presented in Table 2. Furthermore, the table shows sampling times in minutes after waking up in the morning and after daytime naps on both collection days.

**Table 2:** Descriptive statistics of saliva sampling and cortisol values

		Sampling time	Time between wake up and sampling (in min)		Raw cortisol values (nmol/l)
	*N*^a^	M (SD)	M (SD)	*N*^c^	Median (interquartile range)
Out-of-home childcare group* (N = 106)*					
Child woke up; day 1	97	07:37 (0:51)			
Sampling after waking up; day 1	95	08:04 (0:50)	27 (27)	93	9.71 (5.61–13.14)
Child had meal; day 1	93	08:59 (1:23)			
Sampling at 10 a.m.; day 1	97	10:10 (0:26)		96	3.00 (2.22–4.81)
Child had meal; day 1	98	12:55 (1:16)			
Child woke up from the nap; day 1	66	14:20 (0:57)			
Sampling at 2–3 p.m.; day 1	98	14:46 (0:45)	37 (44)^b^	96	2.69 (1.87–4.17)
Child had meal; day 1	97	19:22 (1:04)			
Sampling before sleep; day 1	95	20:10 (0:54)		93	1.01 (0.67–1.98)
Child fell asleep; day 1	96	20:52 (0:57)			
Child woke up; day 2	101	07:01 (0:32)			
Sampling after waking up; day 2	100	07:14 (0:35)	14 (18)	97	8.76 (5.87–12.84)
Child arrived at childcare; day 2	86	08:06 (0:39)			
Child had meal; day 2	80	08:15 (0:27)			
Sampling at 10 a.m.; day 2	96	10:05 (0:17)		99	3.04 (2.28–4.11)
Child had meal; day 2	95	12:02 (1:13)			
Child woke up from the nap; day 2	94	13:47 (0:15)			
Sampling at 2–3 p.m.; day 2	94	14:15 (0:26)	27 (29)^b^	91	4.15 (2.43–6.98)
Child had meal; day 2	96	19:25 (0:58)			
Sampling before sleep; day 2	95	20:11 (0:48)		97	1.04 (0.70–1.69)
Child fell asleep; day 2	97	20:57 (0:50)			
At-home childcare group* (N = 107)*					
Child woke up; day 1	104	07:58 (1:10)			
Sampling after waking up; day 1	103	08:28 (1:06)	30 (34)	100	10.65 (6.22–18.61)
Child had meal; day 1	91	09:17 (1:31)			
Sampling at 10 a.m.; day 1	97	10:25 (0:47)		97	4.65 (3.02–7.02)
Child had meal; day 1	102	12:57 (1:28)			
Child woke up from the nap; day 1	77	14:27 (1:13)			
Sampling at 2–3 p.m.; day 1	103	14:59 (0:55)	37 (42)_b_	98	4.27 (2.39–7.53)
Child had meal; day 1	99	19:27 (1:11)			
Sampling before sleep; day 1	101	20:22 (0:51)		97	1.38 (0.76–3.52)
Child fell asleep; day 1	101	20:53 (2:00)			
Child woke up; day 2	105	07:43 (0:56)			
Sampling after waking up; day 2	103	08:02 (0:54)	20 (17)	96	11.16 (7.60–16.52)
Child had meal; day 2	100	08:55 (1:13)			
Sampling at 10 a.m.; day 2	104	10:25 (1:01)		103	4.03 (2.70–6.20)
Child had meal; day 2	100	12:54 (1:24)			
Child woke up from the nap; day 2	76	14:31 (0:54)			
Sampling at 2–3 p.m.; day 2	102	14:58 (0:51)	33 (34)^b^	99	4.82 (2.68–9.15)
Child had meal; day 2	100	19:39 (1:11)			
Sampling before sleep; day 2	100	20:13 (2:03)		97	1.44 (0.90–3.44)
Child fell asleep; day 2	96	20:49 (2:08)			

^a^Number of subjects with information about their waking, sleeping, and mealtimes

^b^Calculated from the sub-population with reported afternoon naps. A total of 88.7% (*N* = 94) of the children in the out-of-home childcare group took naps on Monday, and 62.3% (*N* = 66) of them took naps on Sunday. Correspondingly, a total of 72% (*N = 77)* of the children in the at-home childcare took naps on Sunday, and 71% (*N* = 76) of the children took naps on Monday

^c^Number of subjects with valid cortisol values, and missing values were caused by failed sampling

### Primary outcomes

*Hypothesis 1* The average diurnal cortisol profiles followed the typical circadian rhythms, where the measured values were highest in the morning after waking up and declined towards the evening being the lowest before going to sleep (Fig. 2, Table 3). The shapes of the diurnal cortisol profiles were also similar between the out-of-home childcare group and the at-home childcare group (Fig. 2). However, the overall saliva cortisol levels (expressed in the original units, nmol/l) were 30% higher (95% CI: [9%, 54%], *p* = 0.004) in the at-home childcare group (Fig. 2; A1, A2, Table 3). This result was also supported by a robust analysis (23% [6%, 42%], *p* = 0.006). A slight increase in the diurnal cortisol pattern was also noticed in both groups and in both measurement days during the afternoon (Fig. 2). This increase was partly explained by the fact that about 35% of the afternoon saliva samples were taken 15–60 min after the daytime naps, which in turn, was found to be associated with 46% higher cortisol levels ([24%, 71%], *p* < 0.0001) compared to cortisol levels measured over 60 min after waking up or measured on children, who had not napped at all.

**Table 3:** The parameter estimates and the corresponding standard errors and *p* values for the fixed effects from the main effects models

Standard analysis					Robust analysis				
Variable	Parameter estimate (*B*)	Standard error	*p*	Relative change (10^*B*)	Variable	Parameter estimate (*B*)	Standard error	*p*^a^	Relative change (10^*B*)
(Intercept)	1.3	0.1	< 0.0001	19.74	(Intercept)	1.08	0.09	< 0.0001	11.91
Group (ref = out-of-home childcare)	0.11	0.04	0.0047	1.30	Group (ref = out-of-home childcare)	0.09	0.03	0.0061	1.23
Day (ref = Sunday)	0.01	0.02	0.4	1.03	Day (ref = Sunday)	0.03	0.01	0.059	1.06
Time spline terms					Time spline terms				
Term 1	− 0.13	0.05	0.014	0.73	Term 1	− 0.15	0.05	0.0023	0.72
Term 2	− 1.35	0.05	< 0.0001	0.04	Term 2	− 1.4	0.04	< 0.0001	0.04
Term 3	− 0.73	0.04	< 0.0001	0.18	Term 3	− 0.81	0.03	< 0.0001	0.15
Nap (ref = no naps/ > 60 min)					Nap (ref = no naps/ > 60 min)				
< 15 min	− 0.03	0.04	0.41	0.93	< 15 min	− 0.02	0.04	0.56	0.95
15–60 min	0.16	0.03	< 0.0001	1.46	15–60 min	0.17	0.03	< 0.0001	1.48
Age	− 0.08	0.04	0.029	0.84	Age	− 0.04	0.03	0.18	0.91
Sex (ref = girl)	0.01	0.04	0.89	1.01	Sex (ref = girl)	0.02	0.03	0.6	1.04
Education (ref = low)					Education (ref = low)				
Mid	− 0.13	0.06	0.016	0.74	Mid	− 0.01	0.05	0.74	0.97
High	− 0.06	0.05	0.24	0.87	High	0.01	0.04	0.79	1.03
Income (ref = low)					Income (ref = low)				
Mid	− 0.07	0.04	0.12	0.86	Mid	− 0.04	0.03	0.28	0.92
High	− 0.01	0.07	0.85	0.97	High	− 0.03	0.06	0.56	0.93
Duration of childcare (years)^b^	− 0.05	0.04	0.25	0.89	Time in childcare (years)^b^	− 0.003	0.04	0.93	0.99
Form of childcare (ref = part-time)^b^	0.05	0.04	0.27	1.12	Form of childcare (ref = part-time)^b^	0.09	0.04	0.015	1.23
Number of children in group^c^	− 0.003	0.006	0.64	0.99	Number of children^c^	− 0.002	0.005	0.69	1.00

The reference classes of the categorical variables are in parenthesis. The response variable is base 10 logarithm of the cortisol level. Therefore, the proportional effect of each predictor on the cortisol level (expressed in the original units, nmol/l) is calculated as 10^*B* (*B* = the unstandardized regression coefficient)

^a^The parameter estimators are assumed to be normally distributed

^b^From the model fitted only to the data on the out-of-home childcare group

^c^From a model fitted to the data on only 96 children (due to some missing data) in the out-of-home childcare group

*Hypotheses 2 and 3* When assessing only the out-of-home childcare group as a whole, the comparison of the afternoon cortisol levels between the out-of-home childcare day and the at-home childcare day (using the interaction model) indicated that the afternoon cortisol levels were 27% ([2%, 57%], *p* = 0.031) higher in the out-of-home childcare day (Fig. 2, B1) compared to the at-home childcare day. However, there was no significant association between cortisol levels and any of the variables: the duration of childcare attendance (*p* = 0.25), the group size in the childcare center (*p* = 0.64), or the childcare form (part-time childcare vs. full-time childcare, *p* = 0.27). Finally, a robust analysis suggested that the children attending the full-time childcare (20 days/month) had 23% higher cortisol levels ([4%; 44%]; *p* = 0.015) than the children attending the part-time care (max. 16 days/month).

*Hypothesis 4* The difference in the afternoon cortisol levels between Monday and Sunday in the at-home childcare group was not significant (20% [−4%, 48%]; *p* = 0.077; Fig. 2, B2)

## Discussion

The present study compared the diurnal saliva cortisol levels between toddlers having out-of-home, center-based childcare and those having at-home, guardian-supervised childcare. To our knowledge, this was the first study to include an at-home, guardian-supervised childcare comparison group. Previous research studies have studied mainly the same children having either an out-of-home childcare day or an at-home childcare day [8, 9, 11, 12]. The at-home childcare comparison group enabled us to explore the functioning of the stress regulation system in the children, who have not yet participated in non-guardian-supervised, out-of-home childcare.

As expected, the shapes of the diurnal cortisol profiles were similar in both groups, i.e., cortisol levels were highest in the morning after waking up and declined towards the evening. Despite the similar diurnal cortisol profiles, the overall cortisol levels were higher in the at-home childcare group in comparison with the out-of-home childcare group (Fig. 2; A1, A2). This result was unexpected, and there might be several plausible explanations for the elevated cortisol levels. To begin with, toddlers in the at-home childcare group had more cortisol values at the higher end of the range in comparison with the out-of-home childcare group. This might derive from a larger variance in the children’s daily rhythms, such as their sleeping, awakening, and meal times, which varied more in the at-home childcare group in comparison with the out-of-home childcare group, where the children followed a more regular rhythm even during their day off (Table 2). It is possible that the regular daily rhythm of the children having out-of-home childcare increases predictability and modifies a child’s cortisol levels as a consequence of habituation to the childcare routines. Previous research studies suggest that repeated exposure to the same type of stressor causes habituation and is related to a decreased HPA axis response [32]. Additionally, maternal prenatal stress may also influence the functioning of the child’s HPA axis and have long-term effects on child outcomes [33, 34]. However, this remains to be investigated in future research.

In addition to the different daily rhythms, maternal educational level was lower in the at-home childcare group. Even though education did not explain the difference between the groups in our statistical models, it may have other unobserved or latent influences on family characteristics. Maternal education is associated with various child outcomes [35], and it may have been linked to factors that could not be controlled for in this study. Earlier research studies suggest that maternal education as well as parental socioeconomic status (SES) correlate with a child’s well-being. Nonetheless, the factors behind these associations are not completely clear [36], and it should be noted that maternal education was generally relatively high in both groups. More detailed data about the family characteristics in both groups could have increased our understanding about the potential sources of a selection effect on these groups.

Among the differences is also the fact that the children in the at-home childcare group were slightly younger than the children in the out-of-home childcare group. Of note, the HPA axis continues to mature during the early childhood years, and earlier studies have suggested an overall decrease in the diurnal cortisol levels by age and across that developmental period [37]. However, a child’s age was controlled for in our statistical models, and therefore we can be quite confident that the age difference between the study participants did not explain the differences in cortisol levels between the groups.

Child temperament may also play an important role in a child’s responses to their environment. One mechanism explaining the relations between temperament and childcare could be the differential susceptibility to environment in children with different temperament phenotypes [38]. A child’s temperament is also an important factor in the child–caregiver interaction. Development may process more smoothly if there is a “goodness of fit” between the caregiver’s practices and the child’s temperamental ability to meet these challenges. On the contrary, there is a “poorness of fit,” if the child cannot fulfill the social expectations due to the temperamental characteristics [39]. In future research studies, this aspect should be studied carefully to fully understand the more nuanced relations between environment and child development.

We were also not able to analyze parent’s attitudes about their employment or their decision to select between out-of-home, center-based childcare and at-home childcare. Despite the good quality of the childcare services in Finland, the at-home childcare rate is a little higher in comparison with other Nordic countries. There is a tendency for some parents to care for their children at home instead of selecting out-of-home childcare, and the government supports financially at-home childcare, until the child is 3 years old [40]. For example, in Denmark, Iceland, Norway, and Sweden, more than 50% of children under 2 years and over 90% of children, aged between 3 and 5 years, regularly have out-of-home childcare. In Finland, only 30% of children under the age of 2 and around 80% of children, aged between 3  and 5 years old, regularly have out-of-home childcare, and children under 1 year old are typically cared for at home [40].

However, all under school age children in Finland have an opportunity to have out-of-home childcare and participate in the ECEC program [22]. Out-of-home childcare is highly regulated and uniform by its characteristics, and the overall educational level of the personnel is high, which may lead to rather homogenous quality within the childcare system. These factors may contribute to the fact that the duration of out-of-home childcare attendance and the group sizes in childcare centers were not associated with cortisol levels in our study sample. This might derive from the limited group sizes and other government-level regulations concerning the out-of-home childcare in Finland.

In summary, it is possible that children cared for at home comprise a more heterogeneous group in comparison with the children attending out-of-home childcare. Some indications of this were the greater variation in daily rhythms and a larger variance in the diurnal cortisol levels between the children in the at-home childcare group. However, it is also possible that the larger variance in cortisol levels was a normal response of the HPA axis to the environmental variance and not necessarily an indication of the higher stress levels, hence, the developmental relevance of these observations needs to be validated during follow-up studies.

Finally, it should be noted that even though there were significant differences in cortisol levels between the groups, the absolute differences in median cortisol values were modest (Table 2), while normal variation in this age group is high [41]. It remains to be proven, whether these elevations in cortisol levels eventually have any effects on child development or later health.

We also analyzed afternoon differences between the days within the groups (Fig. 2; B1, B2). A slight increase in the diurnal cortisol pattern was observed in both groups and in both measurement days in the afternoon (7 h and 10 min since waking up in the morning). This increase was more notable in the out-of-home childcare group during the out-of-home childcare day in comparison with their at-home childcare day (Fig. 2; B1). This finding is in line with earlier research studies [8, 9, 12], which demonstrate higher mid-morning and mid-afternoon cortisol levels in children attending out-of-home childcare. However, in the present study, the afternoon cortisol increase was also observed in the at-home childcare group (Fig. 2; B2) suggesting that regardless of the out-of-home childcare context, afternoons are characterized by increased HPA axis activity. These cortisol increases were partly explained by the afternoon naps, as about 35% of the afternoon saliva samples were taken 15–60 min after waking up. This association between afternoon napping and the cortisol levels was expected, as napping has been found to result in a post-nap cortisol rise in toddlers [21]. Previous research studies have defined the cortisol awakening response (CAR), which describes the period of increased cortisol secretion approximately 30–45 min post-awakening [20]. Most toddlers take naps, and an increase in cortisol values have also been found following both morning and afternoon naps [21].

Nonetheless, the afternoon naps did not explain all of reasons behind the increase in cortisol levels in our study sample. Hence, there are probably other factors than naps also affecting the cortisol increases during the afternoon hours [42]. This raises further questions about whether the afternoon hours are especially demanding for toddlers and thus promote toddlers’ HPA axis activation both in out-of-home childcare and in at-home childcare contexts. The significant increase in the afternoon cortisol levels in children in the out-of-home childcare may also be related to the anticipation of expecting their parents to pick them up. The results of the robust analysis suggest that the children having full-time childcare (20 days/month) had higher cortisol levels compared to children, who had part-time care (max. 16 days/month). This result is in line with earlier research studies indicating that more weekly hours in out-of-home childcare is associated with higher cortisol levels during the out-of-home childcare day and also had carryover effect to days spent at home [14]. Full-time and full-day center-based childcare may be physiologically more demanding for young children than part-time or half-day childcare.

### Limitations and future directions

Despite the large sample size, a novel design (a separate at-home childcare comparison group), several saliva cortisol measurement points spanning 2 consecutive days and many other strengths in this study, there are limitations that should be noted. To begin with, we were not able to analyze the effect of individual parenting methods, child’s attachment to the caregiver or the quality of the out-of-home childcare. Previous research indicates that a secure attachment to the caregiver has potentially a buffering role against stress [43]. Children with a secure attachment to their mother have shown lower cortisol levels during the adaptation phase to childcare in comparison with insecurely attached children [44]. Further, secure attachment to a caregiver in a center-based childcare is associated with decreasing cortisol levels during the childcare day [43]. As parenting style and secure attachment to caregiver [44, 45] as well as childcare quality [43, 46] have been consistently associated with a child’s well-being and HPA axis functioning, it is possible that differences in these domains, which were not measured here, play a role in linking the caregiving environment and diurnal cortisol levels.

We were also not able to gather up-to-date data concerning the family compositions or detailed information about the daily activities during the sample collection days. We collected basic information about the waking, sleeping, and mealtimes as well as the child’s health and medications during the study days. However, there are many other potential variables as well as life events, which may affect a child’s stress regulation both at-home and in an out-of-home childcare environment. Besides maternal prenatal stress, maternal postnatal stress and depression may also influence a child’s stress regulation and well-being [45]. Maternal mental health could also have affected the decision not to work and take care of the child at home instead of selecting out-of-home childcare. In the future, more detailed information about the family circumstances and daily activities in both groups of the children would be useful to increase our understanding about the potential effects of these factors.

Among the limitations is also the fact that saliva samples were collected only during 2 days per group (Sunday and Monday). Cortisol levels may vary from day to day, and normal variation in this age group is large [41]. Finally, the cross-sectional nature of our study design did not enable us to analyze how a child’s age may affect cortisol levels. Earlier research studies suggest a curvilinear developmental course for rising cortisol levels in the center-based, out-of-home childcare settings. That is, increases in cortisol levels across the childcare day appeared to emerge over the infancy period, the levels were highest during the toddler period and decreased by the early school years [47].

## Conclusion

The present study suggests that having out-of-home, center-based childcare is not associated with children’s elevated cortisol levels when contrasted with having at-home guardian-supervised childcare. However, the generalization of these results to different early childhood education contexts should be done cautiously. Children in the at-home childcare group had higher overall cortisol levels in comparison with the children in the out-of-home childcare. Both groups of the children showed increases in the afternoon cortisol levels. This increase was more notable in the out-of-home childcare group during their out-of-home childcare day in comparison with the day they spent at home. This may indicate that the afternoons are particular demanding for toddlers in out-of-home childcare. Hence, the caregivers should consider this when implementing structures and schedules in an out-of-home childcare context. It would also be appropriate for parents to choose part-time care for younger children, if possible. Our results also indicate that the at-home guardian-supervised childcare should hold more attention. For example, the participation in out-of-home childcare could be more strongly recommended for families who are in need of special support. It is also important to recognize children, who are at a higher risk for presenting increased cortisol levels, to improve a caregiver’s ability to support a child’s stress regulation in different situations.

It is possible that various environmental factors as well as a child’s individual characteristic together modifies the stress regulation system. In addition, it should be noted that the overall differences in cortisol levels (nmol/l) were rather small, and individual variation in cortisol concentration between young children is high. Also, we do not have a complete understanding about how the levels of the diurnal cortisol during childhood are related to optimal or non-optimal developmental outcomes later on. These findings call for further research about the factors that influence a child’s HPA axis activity in different childhood environments. Longitudinal research revealing how a child’s age as well as family circumstances and quality of care affect cortisol patterns in different contexts is needed. More research is also needed about the psychological and physiological mechanisms, which affect the vulnerability or resilience to stress on children.
